# Exploring the Therapeutic Potential of Metformin and Vitamin D_3_ Combination in the Management of Diabetes Mellitus

**DOI:** 10.1002/edm2.70384

**Published:** 2026-11-01

**Authors:** Md. Ferdaus Azam, Md. Abu Bakar Siddique Jami, Nafis Yamin Suprio, Md. Ismail Kabir, Sabrina Tasnim Oishi, Jannatul Ferdous Ekra, Nusrat Islam, Samia Zaman, Md. Mofazzal Hossain, Md. Al Amin

**Affiliations:** ^1^ Department of Pharmacy University of Information Technology and Sciences Dhaka Bangladesh; ^2^ Department of Pharmacy Jagannath University Dhaka Bangladesh; ^3^ Department of Pharmacy East West University Dhaka Bangladesh

**Keywords:** diabetes mellitus, hyperglycaemia, metformin, streptozotocin, vitamin D_3_

## Abstract

**Background:**

Diabetes mellitus is a chronic metabolic disorder characterised by hyperglycaemia, dyslipidaemia, hepatic dysfunction, and loss of pancreatic β‐cells. Vitamin D_3_ (D_3_) may influence glucose and lipid metabolism and has been investigated for its potential complementary effects when administered with metformin (MT). In the present study, the anti‐diabetic activity of D_3_ alone and when combined with MT was evaluated in Streptozotocin (STZ) induced diabetic mice.

**Methods:**

Swiss albino mice were made diabetic using STZ (45 mg/kg) injected intraperitoneally for 3 days. Diabetic mice were treated with MT (250 mg/kg), D_3_ (100 μg/kg), or MT + D_3_ for 21 days. The fasting blood glucose, serum lipid profile, body weight, alanine aminotransferase (ALT), and pancreatic histopathology were assessed. The data were analysed by ANOVA and Tukey's post hoc test, and a *p*‐value < 0.05 was considered statistically significant.

**Results:**

The diabetic STZ mice showed sustained hyperglycaemia, weight loss, dyslipidaemia, increased ALT activity and degeneration of the islets of Langerhans. MT and D_3_ treatments were associated with improvements in glycaemic control, and the combination treatment showed the greatest effect. Combined treatment was found to lower the level of total cholesterol (93.06 ± 2.89 mg/dL), LDL (28.66 mg/dL), triglycerides (120.33 ± 2.10 mg/dL) and ALT activity (52.60 ± 3.05 IU/L) and increase HDL (40.33 ± 2.10 mg/dL) when compared with diabetic controls. Moreover, the MT + D_3_ group exhibited significant changes in the total cholesterol/HDL and LDL/HDL ratio and preserved the histoarchitecture of the pancreas.

**Conclusion:**

Metformin and vitamin D_3_, administered individually or in combination, were associated with improvements in several diabetes‐related metabolic parameters. The combination group showed greater observed metabolic outcomes than either treatment alone in STZ‐induced diabetic mice. Further adequately powered preclinical and clinical studies are required to determine the therapeutic relevance of this combination.

## Graphical abstract

Metformin and vitamin D_3_, particularly in combination, improved glycaemic control, lipid profile, liver function, and pancreatic histoarchitecture in STZ‐induced diabetic mice. These findings suggest potential for combination therapy as a complementary approach to diabetes management.

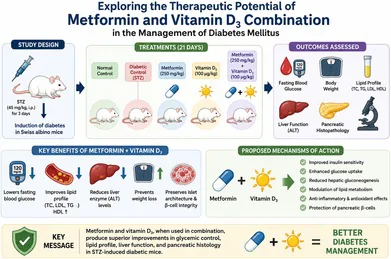

**Abbreviations**
ALT: alanine aminotransferase
ALT/SGPT: alanine aminotransferase/serum glutamic‐pyruvic transaminase
AMPK: AMP‐activated protein kinase
ANOVA: Analysis of variance
AVMA: American Veterinary Medical Association
D_3_
: vitamin D_3_
DC: diabetic control
DM: diabetes mellitus
DNA: deoxyribonucleic acid
FBG: fasting blood glucose
GLP‐1: glucagon‐like peptide‐1
HDL: high‐density lipoprotein
H&E: haematoxylin and eosin
IEAC: Institutional Ethical Approval Committee
IU: international unit
LDL: low‐density lipoprotein
NBF: neutral buffered formalin
NC: normal control
NCG: negative control group
OGTT: oral glucose tolerance test
SGLT‐2: sodium‐glucose cotransporter‐2
SGPT: serum glutamic‐pyruvic transaminase
STZ: streptozotocin
VDR: vitamin D receptor
WHO: World Health Organization

## 1 Introduction

Diabetes mellitus (DM) is a complex group of metabolic disorders characterised by a sustained elevation of blood glucose [1, 2]. DM is one of the most rapidly increasing chronic diseases worldwide [3]. According to the World Health Organization (WHO), DM is responsible for about 4.6 million deaths globally. Also, it is projected to become the sixth leading cause of death by 2030 [4, 5]. DM represents a significant global public health challenge, with more than 90% of the estimated 537 million people living with diabetes in 2021 having Type 2 DM [5]. The high prevalence of DM makes it a serious health issue worldwide, and its long‐term complications like blindness, renal failure, myocardial infarction, amputations and stroke are also a major concern [6].

Common clinical manifestations of DM include polyuria, polydipsia and weight loss, while persistent hyperglycaemia is a defining biochemical feature of the disease [7]. The disease is caused by a lack of insulin production by the pancreas or failure of the body to respond to insulin that is produced [8]. There are a number of therapeutic strategies, drugs and clinical interventions developed to manage DM. The most frequently used drugs for Type 2 DM are insulin sensitizers, insulin secretagogues, α‐glucosidase inhibitors, incretin‐based drugs and sodium‐glucose co‐transporter 2 (SGLT‐2) inhibitors [9].

Streptozotocin (STZ) is a selective β‐cell cytotoxic agent that is used extensively for the induction of experimental diabetes in rodents. STZ‐induced diabetic animals usually exhibit insulin deficiency, hyperglycaemia, polydipsia, and polyuria, which mirror several features of experimental diabetes [10, 11]. STZ‐induced experimental diabetes is widely used to investigate antidiabetic interventions because STZ‐mediated pancreatic β‐cell injury produces persistent hyperglycaemia accompanied by metabolic disturbances, including abnormalities in lipid metabolism. Previous experimental studies have therefore used STZ‐induced diabetic animals to evaluate both glucose‐lowering and lipid‐modulating effects of antidiabetic interventions [10, 12, 13, 14]. However, STZ‐induced diabetes predominantly reflects β‐cell injury and insulin deficiency and does not fully reproduce the complex pathophysiology of human Type 2 diabetes, particularly insulin resistance and the progressive metabolic dysfunction associated with the disease.

Metformin was selected as the reference antidiabetic treatment because of its established glucose‐lowering activity and extensive use in diabetes management. Many previous studies have used metformin as a standard in investigations of antidiabetic properties in streptozotocin‐induced models [12, 13]. Vitamin D_3_ was investigated because of its reported involvement in glucose and lipid metabolism and its potential effects on β‐cell function and metabolic homeostasis. Previous studies have suggested that vitamin D may influence glucose and lipid metabolism and may be associated with pathways related to insulin sensitivity, β‐cell function, oxidative stress, and inflammation [15, 16, 17, 18]. The combination was therefore evaluated to compare the metabolic responses observed following combined administration with those observed following each intervention alone. However, the present study was not designed to formally distinguish additive, synergistic or antagonistic interactions.

Given the limitations associated with current antidiabetic therapies, it is important to explore complementary approaches that may improve glycaemic and metabolic control. Thus, the present study was designed to evaluate the effects of vitamin D_3_ (D_3_) alone and in combination with metformin (MT) on glycaemic and metabolic parameters in STZ‐induced diabetic mice. Although both MT and D_3_ have been reported to exert beneficial metabolic effects, evidence regarding their combined administration in experimental diabetes remains limited, particularly in STZ‐induced diabetic models.

## 2 Materials and Methods

### 2.1 Chemicals

Glucose (Sisco Research Laboratories Pvt. Ltd., India), distilled water, and 70% ethanol for sterilisation following blood collection. A standard glucose assay kit was employed for the estimation of blood glucose levels. Streptozotocin (STZ) extra pure, 98% (Sisco Research Laboratories Pvt. Ltd., India), metformin hydrochloride (Pharma Noble Chem Ltd., Hyderabad, India), and vitamin D_3_ (400 IU/mL, ACI Ltd., Bangladesh).

Citrate buffer was made according to the protocol described by Motyl and McCabe [19], which involves dissolving 1.05 g of citric acid and 1.48 g of sodium citrate in distilled water to get 100 mL of 0.1 M citrate buffer (pH 4.5). 10% neutral buffered formalin (NBF) was prepared using a phosphate‐buffer vehicle and commercial‐grade formaldehyde solution (37%). Mice were anaesthetised for surgery with ketamine (Kain, Renata Pharmaceuticals Limited) and xylazine (A‐Xyla, Acme Laboratories Limited). Commercial reagent kits were used for the determination of serum glutamic‐pyruvic transaminase (SGPT/ALT) and lipid profile analyses as per the manufacturer's instructions. Unless otherwise specified, all chemicals and reagents used in this study were of analytical grade.

### 2.2 Animal Models and Maintenance Conditions

About 70 healthy adult male Swiss albino mice weighing 28 ± 3 g were procured from the International Centre for Diarrhoeal Disease Research, Bangladesh (icddr,b). Male mice were selected in accordance with previous experimental protocols using only male animals in experimental diabetes research [20, 21, 22, 23]. The use of a single sex was also considered to reduce biological variability within the experimental groups. However, this design does not allow assessment of potential sex‐related differences in the metabolic responses.

Animals were kept in polypropylene cages in standard laboratory conditions. The room temperature was kept at 22°C ± 2°C with 55% ± 5% relative humidity under a 12‐h light/dark cycle [24]. The animals could drink tap water at will and were fed a standard pellet diet during the entire experimental period. Mice were acclimatised to the laboratory conditions for 7 days prior to the beginning of the study.

### 2.3 Experimental Design

This study was carried out in three successive stages. During Phase I, normoglycaemic mice (*n* = 30) were subjected to an oral glucose tolerance test (OGTT) to determine baseline glucose tolerance and glycaemic regulation in normal mice. In Phase II, mice (*n* = 30) were fasted overnight and then injected with streptozotocin (STZ) to create experimental diabetes mellitus. The antihyperglycaemic effects of MT, D_3_, and MT + D_3_ combination treatment were assessed in diabetic mice induced by STZ in Phase III over a fixed period of treatment. The blood samples of the mice were taken from the tail vein for biochemical studies.

The experimental design was approved by the University of Information Technology and Sciences (UITS), Department of Pharmacy Ethical Committee (PEC) for Experimentations on Animal, Human, Microbes and Living natural Sources (Reference No.: UITS/PHARM/PEC/2025/09). The study adhered to the ARRIVE 2.0 guidelines and the institutional animal care guidelines. At the end of the experiment, all experimental animals were anaesthetised using halothane vapour (0.05%) followed by cervical dislocation as the method of euthanasia in compliance with the American Veterinary Medical Association (AVMA) Guidelines for the euthanasia of laboratory animals [25].

#### 2.3.1 Oral Glucose Tolerance Test

The mice were subjected to an OGTT to assess glucose tolerance and metabolic response. After 12 h of fasting, the body weight of each mouse was weighed and the baseline blood glucose level was measured using a glucometer (Acuteek Blood Glucose Monitoring System). Six mice per group were given 2 g/kg body weight of glucose orally. The animals were randomly allocated to 5 experimental groups (6 animals per group), including a normal control group, a negative control group, and three groups to be treated with different interventions. Blood glucose was determined at fasting and then at 0, 30, 60, 90, 120, 150 and 180 min after glucose administration [26, 27]. Animals were randomly allocated to the experimental groups. However, no formal a priori sample‐size calculation was performed before initiation of the experiment.

#### 2.3.2 Induction of Experimental Diabetes

Experimental diabetes was induced according to the protocol outlined in a previous study. The STZ solution was prepared in ice‐cold 0.1 M citrate buffer (pH 4.5) just before administration and used immediately to ensure optimum diabetogenic activity [14]. Forty mice were distributed to be induced with diabetes. Group I (*n* = 8) was used as the normal control and administered citrate buffer, Group II (*n* = 32) was administered STZ. Prior to STZ administration, the animals were fasted for 4 h [10]. Diabetes was induced in the animals by injecting STZ (45 mg/kg body weight) intraperitoneally for three consecutive days, while citrate buffer was administered to the control group [28].

Fasting blood glucose (FBG) was determined using tail vein blood samples obtained 5 days after the last STZ injection and a glucometer. Animals with fasting blood glucose levels > 9 mmol/L were classified as diabetic and entered into the treatment phase. In 32 STZ‐treated mice, 25 (78.1%) were successfully induced with diabetes. The diabetic model was successfully established as the fasting blood glucose levels were significantly elevated after STZ administration as compared to the non‐diabetic control group (*p* < 0.001). The induced mice showed a sustained hyperglycaemia typical of STZ‐induced pancreatic β‐cell dysfunction, thus confirming the validity of the model for assessing the anti‐hyperglycaemic activity of MT, D_3_ and their combination.

#### 2.3.3 Evaluation of Hypoglycaemic Activity

Among the 32 STZ‐treated mice, 25 animals developed diabetes successfully. Then 6 non‐diabetic mice from Group I of Phase II were kept as normal control and 24 diabetic mice were randomly divided into 4 groups (Groups II–V; *n* = 6 each). In Phase III, the diabetic animals then received the following treatments orally via gavage for 21 consecutive days [29, 30]. Metformin was administered at a dose of 250 mg/kg body weight [31], while vitamin D_3_ (D_3_) was administered at 100 μg/kg body weight [32]. These doses were selected based on previously reported experimental studies in mouse models using these dose levels.

Group I (normal control): non‐diabetic mice received the vehicle (distilled water).

Group II (diabetic control/negative control): diabetic mice received the vehicle (distilled water).

Group III (metformin‐treated group): Diabetic mice received metformin at a dose of 250 mg/kg and served as the positive control group.

Group IV (combination‐treated group): diabetic mice received metformin (250 mg/kg) in combination with vitamin D_3_ (100 μg/kg). The doses were calculated individually according to the body weight of each animal and administered orally.

Group V (vitamin D_3_‐treated group): diabetic mice received vitamin D_3_ at a dose of 100 μg/kg via oral gavage, with dose volume adjusted according to body weight.

All animals received a standard pellet diet and water ad libitum throughout the 21‐day treatment period. The same duration of oral administration of the vehicle was also given to the normal control group. At the end of the experiment, all of the experimental animals were anaesthetised with halothane vapour (0.05%). Cervical dislocation was used as the method of euthanasia after the animals were anaesthetised. This method followed guidelines from a previous study and adhered to the AVMA guidelines for the euthanasia of laboratory animals.

### 2.4 Biochemical Profiling

Cardiac puncture was performed to collect blood samples, which were then centrifuged at 3000 rpm for 10 min to obtain serum samples [33]. To assess hepatic function, serum alanine aminotransferase (ALT/SGPT) activity was measured using a standard biochemical reagent kit [34]. Total cholesterol, triglycerides, high‐density lipoprotein (HDL) and low‐density lipoprotein (LDL) were measured in serum by standard commercial diagnostic kits (Human GmbH, Germany) according to the manufacturer's instructions.

Blood samples for serum biochemical analyses were collected from three animals per group at the end of the treatment period. Fasting blood glucose was measured by minimally invasive tail‐vein sampling, allowing repeated, non‐terminal measurements in all six animals per group throughout the 21‐day treatment period. In contrast, serum lipid profile and ALT analyses required a larger blood volume obtained by terminal cardiac puncture. Therefore, based on the available experimental resources, these analyses were performed in three animals per group. Thus, the difference in sample size across endpoints resulted from differences in sampling procedures and resource availability rather than animal attrition. Smaller sample sizes may have reduced the statistical power of these biochemical results.

### 2.5 Histopathology Assessment

One mouse from each experimental group was selected for histopathological evaluation. The histopathological assessment was not formally blinded. Following sacrifice, the pancreas was immediately excised and immersed in 10% phosphate‐buffered formaldehyde solution for fixation. The pancreatic tissues were subsequently processed through graded alcohol dehydration and embedded in paraffin wax. The paraffin‐embedded tissues were sectioned at 5 μm and stained with haematoxylin and eosin (H&E). The stained sections were examined under a light microscope at 400× to assess histopathological changes in pancreatic architecture [35]. Because only one representative animal per group was examined, the histopathological assessment was descriptive and qualitative and was not subjected to standardised histopathological scoring or quantitative morphometric analysis.

### 2.6 Statistical Analysis

The data were presented as mean ± standard deviation (SD). Data were analysed statistically with GraphPad Prism (GraphPad Software, USA), version 8.0, and Microsoft Excel 2019 (Microsoft, USA). One‐way analysis of variance (ANOVA) followed by Tukey's post hoc test was used to determine differences between multiple test groups. For parameters measured repeatedly over time, including fasting blood glucose and body weight, two‐way repeated‐measures ANOVA followed by Tukey's post hoc test was applied. The *p*‐value of < 0.05 was regarded as significant.

## 3 Results

### 3.1 Oral Glucose Tolerance Test

The oral glucose tolerance test (OGTT) was used to assess glucose handling and the acute antihyperglycaemic effects of MT, D_3_, and their combination (MT + D_3_). All groups had similar blood glucose levels at baseline (0 min) ranging from 6.23 ± 0.87 to 7.10 ± 1.10 mmol/L, reflecting similar glycaemic status at the time of glucose administration.

After oral glucose loading, blood glucose level was significantly elevated in all glucose groups and peaked at 30 min. The negative control group (NCG) exhibited a peak glucose level of 12.85 ± 3.85 mmol/L, while the MT, D_3_, and MT + D_3_ groups showed peak values of 13.36 ± 3.32, 12.55 ± 2.23, and 12.80 ± 1.60 mmol/L, respectively. After that, blood glucose gradually decreased in all treatment groups, while the NCG had relatively high blood glucose levels throughout the study period (Table 1 and Figure 1).

**TABLE 1:** Oral glucose tolerance test values in experimental mice following treatment with metformin (MT), vitamin D_3_ (D_3_), and their combination (MT + D_3_). Data are presented as mean ± SD (*n* = 6 per group) and expressed in mmol/L.

Time	0 min	30 min	60 min	90 min	120 min	150 min	180 min
NC	6.52 ± 0.8	6.65 ± 0.76	6.42 ± 0.81	6.2 ± 0.55	5.72 ± 0.38	5.66 ± 0.43	5.6 ± 0.33
NCG	7.1 ± 1.1	12.85 ± 3.85	11.1 ± 3.1	9.63 ± 3.43	8.52 ± 2.2	7.76 ± 1.92	7.5 ± 1.55
MT	6.74 ± 1.3	13.36 ± 3.32	8.42 ± 1.92	6.68 ± 0.93	6.28 ± 0.82	5.88 ± 0.68	5.22 ± 0.51
MT + D_3_	6.94 ± 0.6	12.8 ± 1.6	7.52 ± 0.84	5.26 ± 0.44	4.54 ± 0.33	3.8 ± 0.3	3.66 ± 0.36
D_3_	6.23 ± 0.87	12.55 ± 2.23	8.75 ± 1.39	7.61 ± 1.2	6.82 ± 1.03	6.5 ± 1.28	6.18 ± 1.15

Abbreviations: D_3_, vitamin D_3_‐treated group; MT, metformin‐treated group; MT + D_3_, metformin plus vitamin D_3_‐treated group; NC, normal control; NCG, negative control.

**FIGURE 1:**
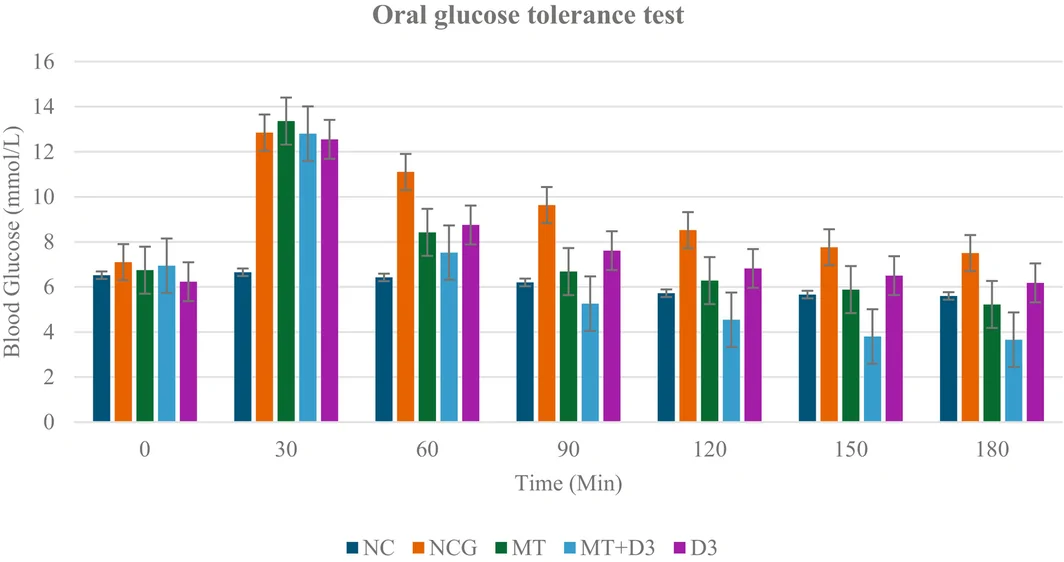
Effect of metformin (MT), vitamin D_3_ (D_3_), and their combination (MT + D_3_) on oral glucose tolerance in Swiss albino mice. Blood glucose concentrations were measured before and at 30, 60, 90, 120, 150 and 180 min after oral glucose administration (2 g/kg body weight). Data are presented as mean ± SD (*n* = 6 per group) and expressed in mmol/L. Statistical significance was considered at *p* < 0.05. D3, vitamin D3‐treated group; MT, metformin‐treated group; MT + D3, metformin plus vitamin D3‐treated group; NC, normal control; NCG, negative control.

The combination‐treated group showed the lowest observed blood glucose concentrations during the later phases of the OGTT. At 60 min, blood glucose levels in the MT + D_3_ group decreased to 7.52 ± 0.84 mmol/L compared with 8.42 ± 1.92 mmol/L in the MT group and 8.75 ± 1.39 mmol/L in the D_3_ group. This trend continued throughout the study period, with the MT + D_3_ group recording the lowest glucose levels at 90, 120, 150, and 180 min (5.26 ± 0.44, 4.54 ± 0.33, 3.80 ± 0.30, and 3.66 ± 0.36 mmol/L, respectively).

At the end of the test (180 min), the MT + D_3_ group exhibited substantially lower glucose levels than the MT group (5.22 ± 0.51 mmol/L), D_3_ group (6.18 ± 1.15 mmol/L), and NCG (7.50 ± 1.55 mmol/L). The glucose levels of the normal control group remained constant during the experiment and returned to near baseline levels at 180 min (5.60 ± 0.33 mmol/L). Overall, the MT + D3 group showed a significantly greater reduction in blood glucose levels during the later phases of the OGTT compared with the monotherapy groups (*p* < 0.05), indicating improved glucose tolerance under the conditions of the present experiment.

### 3.2 Effect of Treatments on Fasting Blood Glucose Levels

The antihyperglycaemic effects of MT, D_3_ and MT + D_3_ were investigated in diabetic mice after 21 days of treatment (Figure 2). The fasting blood glucose level was significantly higher in all diabetic groups than in the NC group at baseline (Day 0). The blood glucose concentration in the diabetic control (DC) group was 11.4 ± 1.36 mmol/L, while in the MT group, it was 12.5 ± 1.47 mmol/L, in the MT + D_3_ group, it was 13.1 ± 1.57 mmol/L, and in the D_3_ group, it was 12.2 ± 1.41 mmol/L, and the blood glucose concentration of the NC group was 7.1 ± 0.45 mmol/L.

**FIGURE 2:**
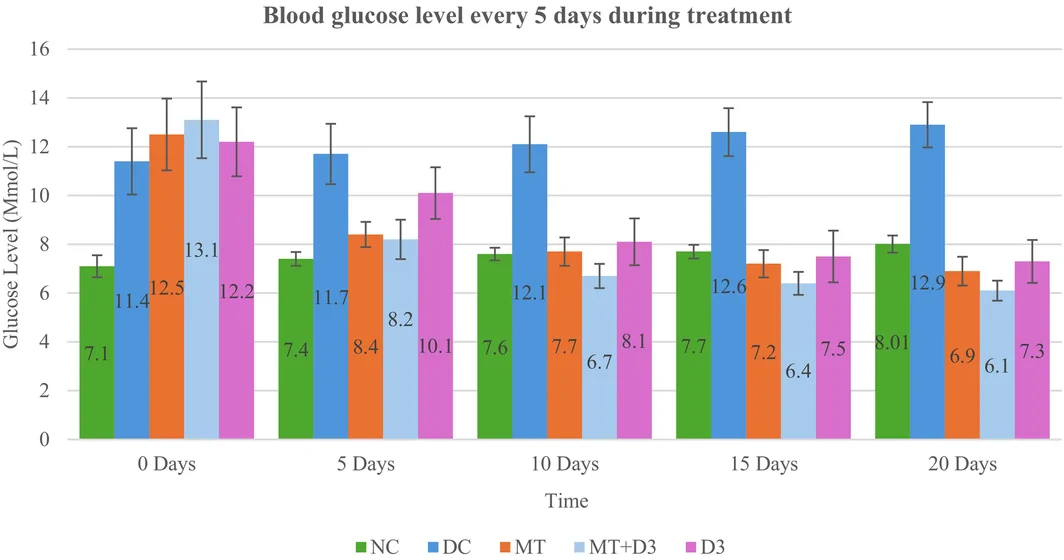
Changes in fasting blood glucose levels during the 21‐day treatment period in streptozotocin (STZ)‐induced diabetic mice treated with metformin (MT), vitamin D_3_ (D_3_), or their combination (MT + D_3_). Fasting blood glucose was measured at baseline (Day 0) and on Days 5, 10, 15, and 20. Data are presented as mean ± SD (*n* = 6 per group) and expressed in mmol/L. Statistical significance was considered at *p* < 0.05. D3, vitamin D3‐treated group; MT, metformin‐treated group; MT + D3, metformin plus vitamin D3‐treated group; NC, normal control; NCG, negative control.

After the start of treatment, blood glucose levels gradually declined in all treatment groups and the DC group remained hyperglycaemic. By Day 5, fasting glucose levels had decreased to 8.4 ± 0.52 mmol/L in the MT group, 10.1 ± 1.06 mmol/L in the D_3_ group, and 8.2 ± 0.81 mmol/L in the MT + D_3_ group. Glucose level in the DC group, however, rose slightly to 11.7 ± 1.24 mmol/L, while the NC group did not change and was 7.4 ± 0.28 mmol/L.

On Day 10, there was a further decrease in blood glucose levels. The MT + D_3_ group exhibited the lowest glucose level (6.7 ± 0.50 mmol/L), followed by the MT group (7.7 ± 0.58 mmol/L) and the D_3_ group (8.1 ± 0.96 mmol/L). The glucose levels in the DC group were still high (12.1 ± 1.15 mmol/L), reflecting ongoing diabetes.

All treated groups showed glucose‐lowering effect by Day 15. The MT + D_3_ group had the lowest fasting glucose level (6.4 ± 0.47 mmol/L) than the MT group (7.2 ± 0.56 mmol/L) and the D_3_ group (7.5 ± 1.06 mmol/L). The glucose level in the DC group rose further to 12.6 ± 0.98 mmol/L while that of the NC group remained in the normal physiological range (7.7 ± 0.28 mmol/L).

The MT + D_3_ group showed the lowest fasting blood glucose (FBG) level at the end of the treatment period (Day 20) at 6.1 ± 0.41 mmol/L. This value was lower than that observed in the MT group (6.9 ± 0.59 mmol/L) and the D_3_ group (7.3 ± 0.88 mmol/L). The glucose level was significantly higher in the DC group (12.9 ± 0.93 mmol/L) compared to the NC group (8.01 ± 0.35 mmol/L), which was close to the normal range. The MT + D_3_, MT and D_3_ groups had blood glucose levels that were about 53.4%, 44.8% and 40.2% lower than baseline levels. FBG level in all the treatment groups significantly decreased from the diabetic control group during the study period (*p* < 0.05).

### 3.3 Effect of Treatments on Body Weight

Weight loss was measured at 5‐day intervals for 21 days during treatment with MT, D_3_, and MT + D_3_ to evaluate the weight loss associated with diabetes (Figure 3). The body weights were similar between the experimental groups at baseline (Day 0), ranging from 26.4 ± 1.60 g for the NC group to 29.0 ± 1.94 g for the MT group.

**FIGURE 3:**
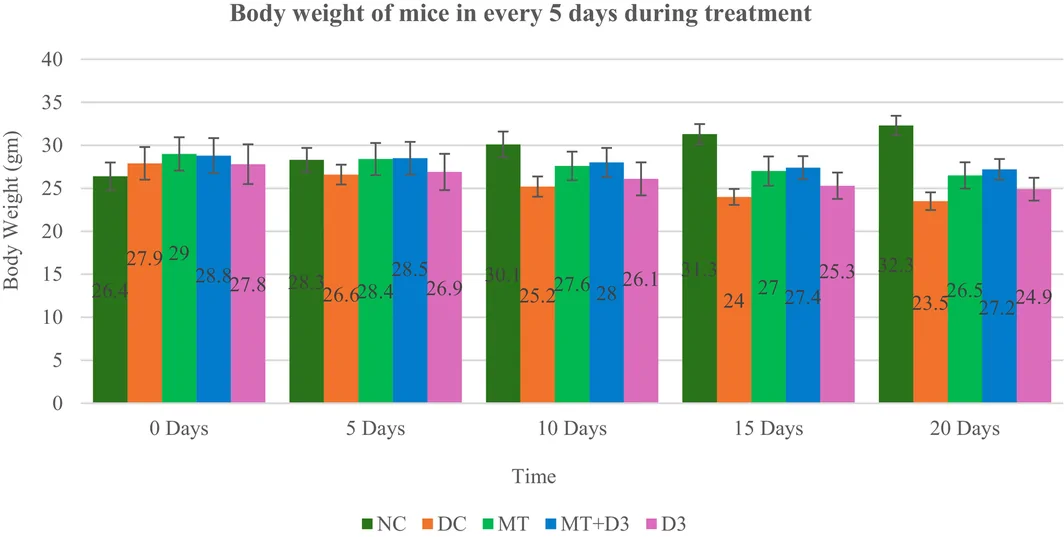
Changes in body weight during the 21‐day treatment period in streptozotocin (STZ)‐induced diabetic mice treated with metformin (MT), vitamin D_3_ (D_3_), or their combination (MT + D_3_). Body weight was recorded at baseline (Day 0) and at 5‐day intervals through Day 20. Data are presented as mean ± SD (*n* = 6 per group) and expressed in grams. Statistical significance was considered at *p* < 0.05. D3, vitamin D3‐treated group; MT, metformin‐treated group; MT + D3, metformin plus vitamin D3‐treated group; NC, normal control; NCG, negative control.

In NC group, the body weight increased steadily and by Day 20 it measured 32.3 ± 1.13 g, which was within the normal range of growth and development. DC group, however, experienced a gradual loss of body weight from 27.9 ± 1.90 g to 23.5 ± 1.03 g on Day 20, which is an approximate loss of 15.8%.

MT, D_3_ and MT + D_3_ treatment reduced diabetes‐associated weight loss to varying extents. The MT‐treated group showed a gradual decline in body weight from 29.0 ± 1.94 g to 26.5 ± 1.53 g by Day 20, whereas the D_3_‐treated group decreased from 27.8 ± 2.31 g to 24.9 ± 1.33 g over the same period. The body weight loss was the lowest in the combination‐treated group, which started at 28.8 ± 2.04 g and ended at 27.2 ± 1.21 g on Day 20.

At the end of the treatment period, the MT + D_3_ group had higher body weight than both the MT‐treated and D_3_‐treated groups, and was still significantly higher than the diabetic control group. The MT + D_3_ group lost about 5.6% of their body weight compared with the baseline weight, whereas the MT and D_3_ groups lost 8.6% and 10.4% of their baseline body weight, respectively. The results showed that MT and D_3_, especially in combination, were able to reduce body weight loss and improve the overall metabolic status of the diabetic mice compared to untreated diabetic mice (*p* < 0.05).

### 3.4 Histopathological Evaluation of Pancreatic Tissue

To qualitatively evaluate diabetes‐induced morphological changes in islets of Langerhans and the possible protective effect of MT, D_3_ and their combination, histopathological examination of pancreatic tissue was performed (Figure 4). Haematoxylin and eosin (H&E) staining was performed on representative pancreatic sections from each experimental group, and a light microscope was used to examine them. Because the histopathological assessment was based on one representative animal per group and was not subjected to quantitative scoring or morphometric analysis, these observations are descriptive and should not be interpreted as statistically validated evidence of treatment‐associated pancreatic protection.

**FIGURE 4:**
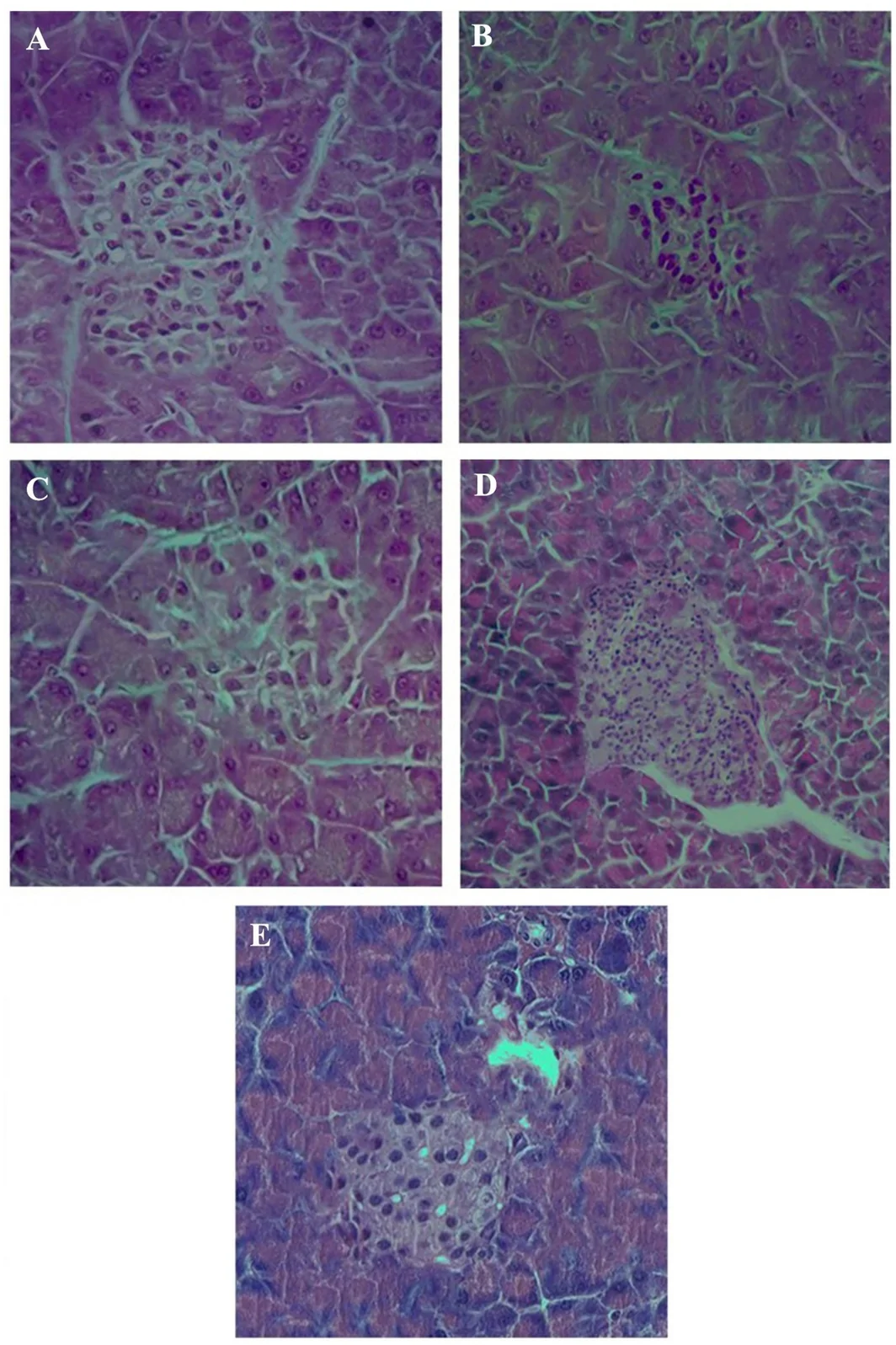
Representative histological appearance of pancreatic tissue from streptozotocin (STZ)‐induced diabetic mice following treatment with metformin (MT), vitamin D3, or their combination. Pancreatic sections were stained with haematoxylin and eosin (H&E) and examined by light microscopy. (A) Normal control (NC); (B) diabetic control (DC); (C) metformin‐treated group (MT); (D) metformin plus vitamin D3‐treated group (MT + D3); and (E) vitamin D3‐treated group (D3). Images represent one representative animal from each group and are presented for qualitative morphological assessment. Images were captured at 400× magnification.

In the NC group, the histoarchitecture of the pancreas showed a normal islet of Langerhans surrounded by organised exocrine pancreatic tissue. There was no evidence of cellular degeneration, inflammatory infiltration, or structural abnormalities (Figure 4A).

However, pancreatic tissue from the DC group exhibited significant histopathological changes such as islet shrinkage and disorganisation, loss of normal cellular structure, and decrease in islet size, suggesting that the STZ had caused extensive damage to the pancreas (Figure 4B). These observations are similar to those of the β‐cell injury that occurs in experimental diabetes.

The MT‐treated group exhibited partial restoration of the pancreatic morphology and showed better islet structure and cellular organisation than the diabetic control group (Figure 4C). Likewise, D_3_ treatment resulted in moderate recovery of the pancreatic architecture with partial restoration of islet size and tissue organisation (Figure 4E).

Among the representative sections examined, the MT + D_3_ group showed comparatively better‐preserved pancreatic histoarchitecture than the diabetic control and monotherapy groups. The islets of Langerhans were relatively well preserved and showed a close resemblance to the normal control islets, and less disruption of the structures than the diabetic control islets, with better organisation of the cells (Figure 4D). The MT + D_3_ group showed comparatively better‐preserved pancreatic histoarchitecture. However, when interpreting the results, it should be taken into account that the assessment was qualitative and based on one representative animal per group.

### 3.5 Biochemical Parameters

#### 3.5.1 Total Cholesterol

Diabetes associated dyslipidaemia was seen as the serum total cholesterol level was significantly elevated in DC group (161.33 ± 9.03 mg/dL) compared to NC group (87.66 ± 5.03 mg/dL) (Figure 5). MT treatment resulted in a significant decrease in total cholesterol to 112.00 ± 6.00 mg/dL (*p* < 0.01 vs. DC); and D_3_ treatment resulted in a moderate decrease in total cholesterol to 143.00 ± 7.54 mg/dL (*p* < 0.05 vs. DC). The MT + D_3_ group showed the lowest observed total cholesterol level among the treatment groups, bringing total cholesterol levels down to 93.06 ± 2.89 mg/dL, which was similar to the NC group and lower than the DC and D_3_ groups (*p* < 0.001). Relative to diabetic control, the combination therapy resulted in a 42.3% decrease in serum total cholesterol, whereas the MT and D_3_ monotherapy groups resulted in a 30.6% decrease and 11.4% decrease respectively (Figure 5A).

**FIGURE 5:**
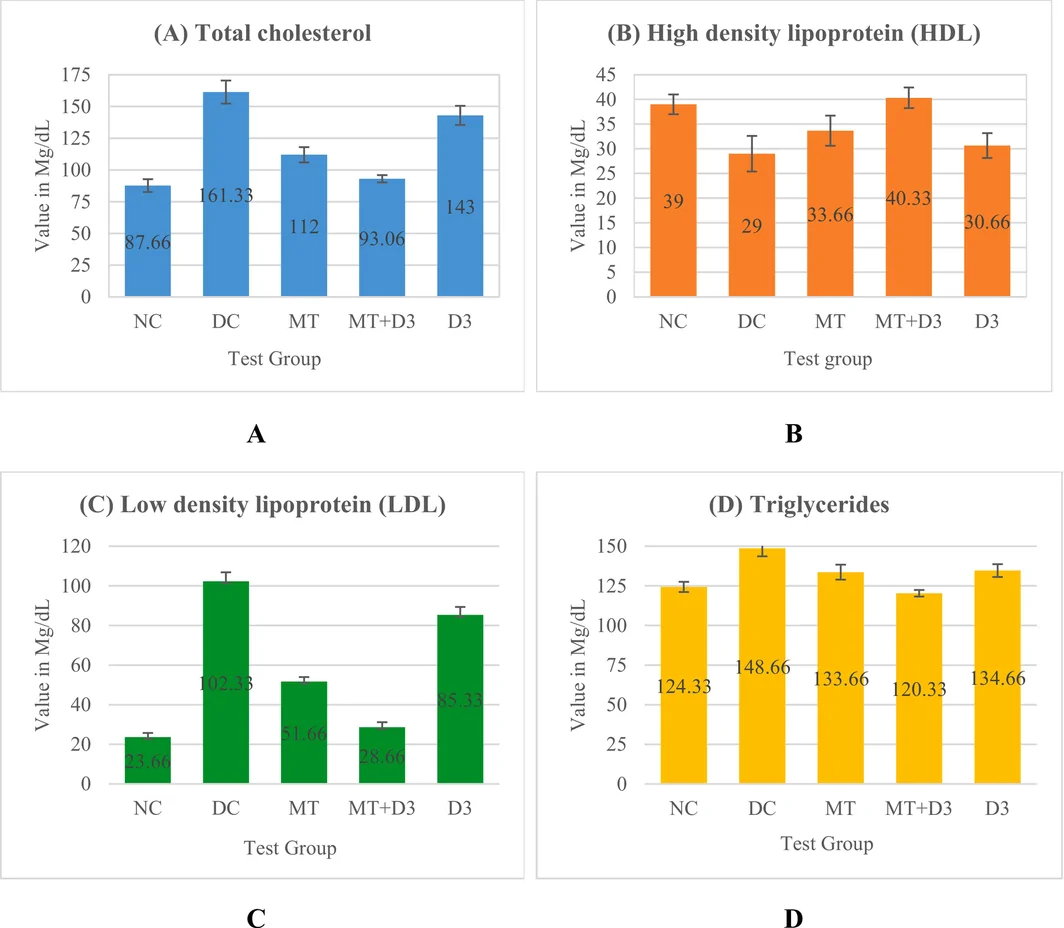
Effects of metformin (MT), vitamin D_3_ (D_3_), and their combination (MT + D_3_) on serum lipid parameters in streptozotocin (STZ)‐induced diabetic mice. (A) Total cholesterol, (B) high‐density lipoprotein (HDL), (C) low‐density lipoprotein (LDL), and (D) triglycerides. Data are presented as mean ± SD (*n* = 3 per group) and expressed in mg/dL. Statistical significance was considered at *p* < 0.05. D3, vitamin D3‐treated group; MT, metformin‐treated group; MT + D3, metformin plus vitamin D3‐treated group; NC, normal control; NCG, negative control.

#### 3.5.2 High‐Density Lipoprotein (HDL)

Serum HDL had remarkably decreased in the DC group (29.00 ± 3.60 mg/dL) as compared with the NC group (39.00 ± 2.00 mg/dL) (*p* < 0.05), indicative of impaired lipid metabolism. MT led to a significant rise in HDL levels to 33.66 ± 3.05 mg/dL, while D_3_ showed a slight increase to 30.66 ± 2.51 mg/dL. The MT + D_3_ showed the highest observed HDL level among the treatment groups, raising HDL levels to 40.33 ± 2.10 mg/dL, which was significantly higher than in the DC, MT, and D_3_ groups (*p* < 0.05) and was comparable to the NC group. The HDL level was found to be about 39.1% higher in the combination‐treated group than the diabetic control group, whereas 16.1% and 5.7% higher were seen in the MT and D_3_ groups, respectively. Combination therapy generates an elevation of HDL, which is significant, suggesting a better lipid profile (Figure 5B).

#### 3.5.3 Low‐Density Lipoprotein (LDL)

The diabetic control (DC) group had a significantly higher level of LDL in the serum than the normal control (NC) group, showing that dyslipidaemia associated with diabetes is quite severe (102.33 mg/dL compared to 23.66 mg/dL in the NC group). The treatment with MT significantly lowered the LDL levels (*p* < 0.01 vs. DC) to 51.66 mg/dL, while the treatment with vitamin D_3_ alone had a moderate effect that lowered the LDL levels to 85.33 mg/dL (*p* < 0.05 vs. DC). MT and D_3_ showed the most lipid‐lowering effect with the level of LDL dropping to 28.66 mg/dL, close to the NC group, but significantly less than the DC and D_3_ treated groups (*p* < 0.001). The LDL levels were reduced by approximately 72.0%, 49.5%, and 16.6% in the MT + D_3_, MT, and D_3_ groups, respectively, compared to the diabetic control group (Figure 5C).

#### 3.5.4 Triglycerides

Diabetic control mice had significantly higher levels of serum triglycerides (148.66 ± 5.03 mg/dL) than the normal controls (124.33 ± 3.21 mg/dL) (*p* < 0.05), indicating development of diabetic dyslipidaemia. Treatment with MT lowered triglycerides to 133.66 ± 4.72 mg/dL, whereas D_3_ treatment did so to a similar extent (134.66 ± 4.04 mg/dL). MT + D_3_ combination group showed the lowest observed results, having triglyceride level 120.33 ± 2.10 mg/dL which was significantly lower than the DC group (*p* < 0.001) and slightly lower than the NC group. The triglyceride level was reduced by about 19.1% in the combination treated group and 10.1% and 9.4% in the MT and D_3_ alone groups, respectively, when compared with diabetic controls (Figure 5D).

#### 3.5.5 Total Cholesterol / HDL Ratio

A significant difference was observed between the diabetic control (DC) and normal control (NC) groups in terms of the total cholesterol/HDL ratio, which is an important factor of cardiovascular risk, with a significantly higher ratio in the DC group (5.59 ± 0.37) than in the NC group (2.25 ± 0.02), indicating severe lipid imbalance due to diabetes. The ratio is significantly improved with metformin treatment (MT) (3.33 ± 0.13, *p* < 0.01 vs. DC) but moderately with vitamin D_3_ treatment (4.66 ± 0.14, *p* < 0.05 vs. DC). MT + D_3_ combination group exhibited the most remarkable reduction in the ratio (2.31 ± 0.11), which was not significantly different from the NC group, but was significantly lower than those of the DC and D_3_‐treated groups (*p* < 0.001). This ratio was lowered by around 58.7% in the combination‐treated group, showing a significant improvement in lipid homeostasis when compared to the diabetic control group.

#### 3.5.6 LDL/HDL Ratio

Diabetic control mice had a significantly higher LDL/HDL ratio (3.55 ± 0.28) compared to normal mice (0.60 ± 0.04). Administration of MT significantly decreased the ratio to 1.54 ± 0.09 (*p* < 0.01 vs. DC), while D_3_ treatment reduced the ratio to 2.79 ± 0.10 (*p* < 0.05 vs. DC). Remarkably, MT + D_3_ significantly reduced the LDL/HDL ratio to 0.71 ± 0.08, which was comparable to the NC group and significantly lower than the MT and D_3_ monotherapy groups (*p* < 0.001). The LDL/HDL ratio was reduced by 80.0% in the combination‐treated group; both MT and D_3_ groups reduced the ratio by 56.6% and 21.4%, respectively, when compared to the diabetic control group. The improved ratio further confirms that the combined MT and D_3_ treatment showed the largest observed reduction in the LDL/HDL ratio among the treatment groups. However, no direct cardiovascular outcomes were assessed.

### 3.6 Evaluation of Liver Function

The activity of the serum alanine aminotransferase (ALT/SGPT) was measured to assess the hepatic function and degree of liver damage caused by STZ‐induced diabetes. The diabetic control (DC) group showed a significantly higher level of ALT (89.60 ± 5.68 IU/L) than the normal control (NC) group (40.30 ± 4.16 IU/L), suggesting a greater degree of hepatic stress and dysfunction due to chronic hyperglycaemia (*p* < 0.001). Metformin (MT) treatment significantly lowered ALT activity to 63.30 ± 3.21 IU/L (*p* < 0.01 vs. DC), whereas treatment with vitamin D_3_ moderately lowered ALT activity to 71.00 ± 3.60 IU/L (*p* < 0.05 vs. DC) (Figure 6).

**FIGURE 6:**
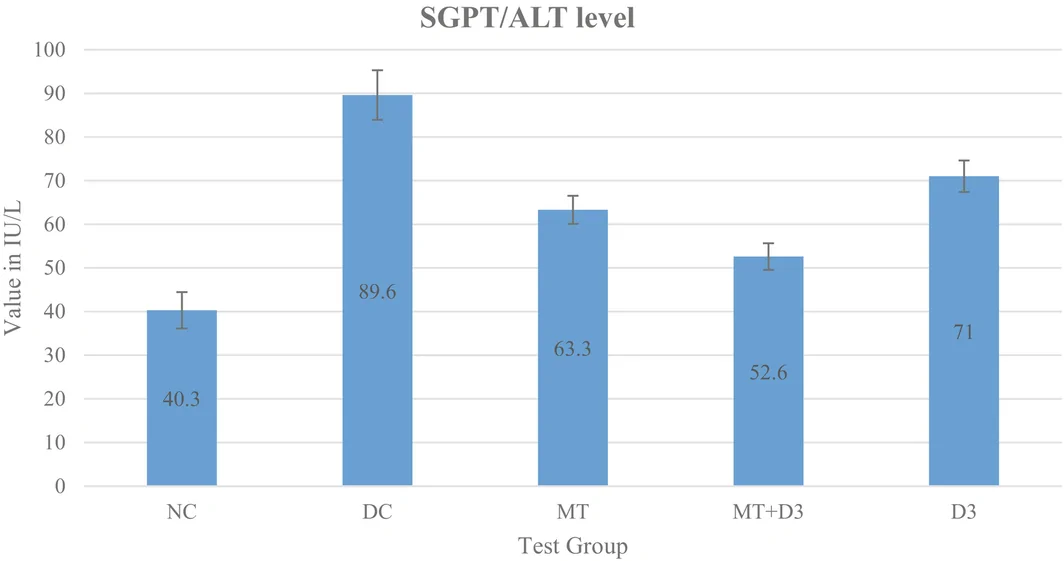
Effects of metformin (MT), vitamin D_3_ (D_3_), and their combination (MT + D_3_) on serum alanine aminotransferase (ALT/SGPT) activity in streptozotocin (STZ)‐induced diabetic mice. Data are presented as mean ± SD (*n* = 3 per group). Statistical significance was considered at *p* < 0.05. D3, vitamin D3‐treated group; MT, metformin‐treated group; MT + D3, metformin plus vitamin D3‐treated group; NC, normal control; NCG, negative control.

The MT + D_3_ group showed the lowest observed ALT activity (52.60 ± 3.05 IU/L) compared to the other treatment groups (DC, MT and D_3_) (*p* < 0.001). In the diabetic control group, the reduction in ALT activity was around 29.4% and 20.8% in the MT and D_3_ monotherapy groups, respectively, while the combination therapy group saw a decrease of about 41.3% in ALT activity. While the ALT levels are still elevated in the combination‐treated group compared to normal controls, the significant decrease suggests that there is significant amelioration of diabetes‐induced liver damage. The combination group showed the greatest observed reduction in ALT activity among the treatment groups. However, because only ALT was assessed and additional hepatic biomarkers or liver histology were not evaluated, this finding should be interpreted as an improvement in diabetes‐associated ALT elevation rather than definitive evidence of hepatoprotection.

## 4 Discussion

The present study investigated the antidiabetic effects of vitamin D_3_, administered alone or in combination with metformin, in streptozotocin‐induced diabetic mice. The OGTT showed comparable baseline glucose levels among the groups, indicating similar glucose handling before treatment. Following STZ administration, the diabetic mice developed persistent hyperglycaemia, confirming successful establishment of the experimental diabetes model. STZ induces pancreatic β‐cell injury through mechanisms involving DNA alkylation and oxidative stress, resulting in impaired insulin availability and hyperglycaemia [10, 14]. During the treatment period, both MT and D_3_ reduced blood glucose levels, while the MT + D_3_ combination produced the greatest reduction. Metformin improves glucose homeostasis mainly through suppression of hepatic glucose production and improvement of insulin sensitivity, partly involving AMPK‐related pathways [36, 37]. Vitamin D_3_ may influence glucose metabolism through VDR‐mediated signalling and calcium‐dependent mechanisms involved in β‐cell function and insulin secretion [38]. The greater glycaemic improvement observed with the combination may therefore reflect complementary effects of the two treatments. However, these molecular pathways were not directly examined in the present study, and the observed response should not be interpreted as evidence of confirmed AMPK or VDR activation.

Body weight changes further reflected the metabolic status of the animals. Diabetic control mice showed progressive body weight loss, whereas MT and D_3_ treatment partially attenuated this effect. The greatest observed preservation of body weight occurred in the MT + D_3_ group. This improvement may be associated with better glycaemic control and improved nutrient utilisation during treatment. Similar effects have been reported in experimental diabetic models treated with metformin and vitamin D_3_ [39].

Histopathological examination of pancreatic tissue provided supportive qualitative evidence of treatment‐associated morphological changes. Degeneration of pancreatic islets, reduced cellular density, and structural disorganisation were observed in diabetic control animals, consistent with STZ‐associated pancreatic injury. In contrast, representative pancreatic sections from the MT + D_3_‐treated group exhibited relatively improved islet morphology and preservation of cellular architecture. These findings may indicate reduced pancreatic morphological damage following treatment; however, the present histopathological assessment was qualitative and based on one representative animal per group. Therefore, the findings cannot establish quantitative preservation or regeneration of pancreatic β‐cells. Previous studies have reported that vitamin D_3_ may influence oxidative stress, inflammation, and β‐cell survival in pancreatic tissue [40]. Metformin may also indirectly support pancreatic function by improving glycaemic control and reducing metabolic stress associated with chronic hyperglycaemia [41]. However, because serum insulin, β‐cell mass, insulin immunohistochemistry, oxidative stress markers, and inflammatory mediators were not assessed, the present findings cannot establish whether the observed morphological differences were associated with improved β‐cell function or specific molecular mechanisms.

The diabetic control group also exhibited marked dyslipidaemia, characterised by increased total cholesterol, LDL, triglycerides, and atherogenic lipid ratios together with reduced HDL. MT and D_3_ individually improved several lipid parameters, whereas the MT + D_3_ group generally showed the largest observed improvements in the measured lipid parameters and resulted in values approaching those of the normal control group. These findings are consistent with previous reports describing lipid‐modulating effects of metformin and vitamin D_3_, potentially related to improved metabolic regulation, lipid metabolism, and inflammatory processes [30, 42, 43]. The improved lipid ratios indicate a more favourable atherogenic lipid profile. However, cardiovascular risk itself was not directly assessed in the present study.

Hepatic dysfunction is another metabolic consequence that may occur in diabetes and can be associated with alterations in serum liver enzymes. Diabetic control mice also showed increased serum ALT activity, indicating diabetes‐associated hepatic stress or dysfunction. Both MT and D_3_ reduced ALT levels, with the greatest reduction observed in the MT + D_3_ group. These findings are consistent with previous reports of beneficial effects of metformin and vitamin D_3_ on hepatic abnormalities associated with diabetes [44]. However, because only ALT was measured and hepatic histology or additional liver‐function markers were not assessed, the findings should be interpreted as improvement in diabetes‐associated ALT elevation rather than definitive evidence of hepatoprotection.

Overall, the MT + D_3_ group showed the greatest observed improvements across several measured outcomes, including blood glucose, body weight, lipid parameters, and ALT activity. The qualitative histopathological observations also showed comparatively better‐preserved pancreatic morphology in the representative section examined. The greater response may reflect complementary biological actions of the two agents. Nevertheless, the present study was not designed to establish pharmacological additivity or synergy, and direct statistical superiority of the combination over metformin monotherapy was not consistently established across all endpoints. Therefore, the observed differences should be interpreted as experimental observations rather than definitive evidence of an incremental therapeutic advantage or a confirmed synergistic interaction.

The STZ‐induced model is useful for investigating β‐cell injury, hyperglycaemia, and diabetes‐associated metabolic abnormalities. However, STZ‐induced diabetes does not fully reproduce the complex pathophysiology of human Type 2 diabetes, particularly insulin resistance, obesity, and progressive β‐cell dysfunction [45]. Insulin sensitivity and insulin resistance were also not directly assessed, limiting interpretation of the metabolic effects in relation to insulin sensitivity. In addition, the present study did not measure serum insulin, HbA1c, circulating vitamin D status, oxidative stress biomarkers, calcium/renal safety, food and water intake, inflammatory mediators, or molecular markers of glucose‐signalling pathways. HbA1c was not measured, so the study does not directly assess longer‐term glycaemic exposure. Therefore, the mechanisms underlying the observed effects cannot be established from the current data. Future studies should include daily or periodic measurement of food and water consumption to determine whether treatment‐related metabolic effects are independent of changes in intake. An additional limitation is that direct statistical superiority of the MT + D_3_ combination over metformin monotherapy was not consistently established or reported across all measured endpoints. Because only male animals were included, the present study cannot determine whether sex‐related differences influence the metabolic responses.

The histopathological assessment was qualitative and based on one representative animal per group, without standardised histopathological scoring or quantitative morphometric analysis. A priori power calculation was not performed before the original experiment, and a formally blinded histopathological assessment was not documented. The absence of an a priori power calculation limits the ability to determine whether the study was adequately powered to detect prespecified effects, particularly for the biochemical endpoints assessed in three animals per group. These factors may have limited statistical power and introduced potential observer‐related bias. Furthermore, only three animals per group were used for serum lipid and ALT analyses, which may have limited the statistical power of these endpoints. The relatively small number of animals in each experimental group also limits the statistical power and generalisability of the findings. The 21‐day treatment period was relatively short and did not allow assessment of the long‐term efficacy or safety of the combined treatment. Therefore, the present findings should be considered preliminary and interpreted within the context of these methodological limitations. Future research should extend the study duration beyond 21 days to determine the sustainability of the observed metabolic benefits.

The present study also did not include a formal pharmacological interaction analysis. Although the combination‐treated group showed greater improvements in several measured outcomes than either monotherapy, the experimental design was not specifically structured to determine whether the combined response was additive, synergistic, or antagonistic. Formal interaction analysis using an appropriate factorial experimental design would be required to establish pharmacological synergy. Thus, the greater response observed with MT + D_3_ should be regarded as an experimental observation rather than evidence of confirmed synergistic interaction. Serum calcium and renal function parameters were not assessed, so the present study does not establish the systemic safety of repeated vitamin D_3_ administration. Also, hepatic biomarkers, including AST, ALP, bilirubin, and hepatic histology, were not assessed in this study. Therefore, future research should keep these in focus and these should also be taken into account while interpreting the results. In addition, although the combination‐treated group showed the greatest observed improvements in several outcomes, direct statistical superiority of MT + D_3_ over metformin monotherapy was not consistently established or reported for all endpoints. Therefore, the present findings should not be interpreted as definitive evidence that vitamin D_3_ provides an incremental therapeutic benefit beyond metformin alone. Also, further validation in high‐fat diet/insulin‐resistant or other Type 2 diabetes models can be an important direction for future research.

The original detailed statistical output and individual‐level datasets were not retained. Therefore, some exact *p*‐values could not be reconstructed retrospectively, and results are reported using conventional threshold levels based on the available original analyses. In addition, the present findings provide preliminary experimental evidence that the combined administration of vitamin D_3_ and metformin was associated with favourable changes in several measured metabolic outcomes in STZ‐induced diabetic mice. The combination showed the largest observed improvements in glycaemic control, preservation of body weight, pancreatic morphology, lipid abnormalities, and ALT activity among the treatment groups. However, the absence of mechanistic biomarkers, quantitative histopathological assessment, formal interaction analysis, and long‐term evaluation limits definitive interpretation of these findings. Larger studies incorporating adequate sample sizes, quantitative histopathological assessment, insulin and HbA1c measurements, vitamin D status, oxidative and inflammatory biomarkers, molecular pathway analysis, longer treatment periods, formal interaction analysis, and alternative diabetes models are needed to confirm the reproducibility, mechanisms, long‐term safety, and potential translational relevance of the metformin–vitamin D_3_ combination.

## 5 Conclusion

The present study demonstrated that metformin and vitamin D_3_, administered individually or in combination, improved several diabetes‐associated metabolic abnormalities in STZ‐induced diabetic mice. The metformin and vitamin D_3_ combination group showed the greatest observed improvements in several measured outcomes, including glycaemic parameters, body weight, lipid parameters, and serum ALT activity, while representative pancreatic histology showed comparatively better preservation of tissue architecture. However, the present study does not establish a synergistic or additive interaction between metformin and vitamin D_3_, nor does it determine the molecular mechanisms responsible for the observed differences. Also, direct statistical superiority over metformin monotherapy was not consistently established across all endpoints. These findings should consequently be considered preliminary experimental evidence. Further studies incorporating insulin and HbA1c measurements, mechanistic biomarkers, quantitative histopathological assessment, formal drug‐interaction analysis, longer treatment periods, and insulin‐resistant/Type 2 diabetes models are warranted before the therapeutic relevance of vitamin D_3_ as an adjunct to metformin can be established.

## Author Contributions

**Md. Ferdaus Azam:** methodology, conceptualization, formal analysis, writing – original draft, supervision, project administration, visualization, data curation. **Md. Abu Bakar Siddique Jami:** software, data curation, writing – original draft, investigation, formal analysis, visualization, writing – review and editing. **Nafis Yamin Suprio:** investigation, writing – original draft. **Md. Ismail Kabir:** investigation, writing – original draft. **Sabrina Tasnim Oishi:** investigation, writing – original draft. **Jannatul Ferdous Ekra:** investigation, writing – original draft. **Nusrat Islam:** investigation, writing – original draft. **Samia Zaman:** investigation, writing – original draft. **Md. Mofazzal Hossain:** validation, resources, supervision, writing – review and editing. **Md. Al Amin:** writing – review and editing, validation, formal analysis, writing – original draft, data curation.

## Funding

The authors have nothing to report.

## Disclosure

During the preparation of this manuscript, the authors used Grammarly for Windows (Version 1.2.211) to improve the grammar, readability, and language of the manuscript. Additionally, ChatGPT (GPT‐5.2) was used for preparing the graphical abstract. The tools were not used for study design, data analysis, result interpretation, or the generation of scientific content. All suggestions were carefully reviewed and validated by the authors against the original data and relevant literature, and the authors take full responsibility for the final content. No confidential, proprietary, or identifiable human subject data were entered into the software.

## Ethics Statement

The experimental procedure was approved for animal model study by the Department of Pharmacy Ethical Committee (PEC), University of Information Technology and Sciences (Reference No.: UITS/PHARM/PEC/2025/09). All experimental procedures involving animal subjects were carried out in accordance with relevant institutional and international guidelines and regulations.

## Conflicts of Interest

The authors declare no conflicts of interest.

## Data Availability

All data generated or analysed during this study are included in this published article.
